# Physical Inactivity and Related Barriers: A Study in a Community Dwelling of Older Brazilians

**DOI:** 10.1155/2012/685190

**Published:** 2012-11-06

**Authors:** Sebastião Gobbi, Emerson Sebastião, Camila Bosquiero Papini, Priscila Missaki Nakamura, Américo Valdanha Netto, Lilian Teresa Bucken Gobbi, Eduardo Kokubun

**Affiliations:** ^1^Department of Physical Education, Universidade Estadual Paulista (UNESP), Avenida 24-A, 1515 Bela Vista, 13506-900 Rio Claro, SP, Brazil; ^2^Department of Kinesiology and Community Health, University of Illinois at Urbana-Champaign, 906 South Goodwin Avenue, Urbana, IL 61801, USA

## Abstract

This study sought to investigate the prevalence of physical inactivity and related barriers in older Brazilian adults. A cross-sectional, population-based study was conducted, and a stratified random sampling procedure was used. A total of 359 older adults were interviewed. The long version of the International Physical Activity Questionnaire (IPAQ) and the Questionnaire of Barriers to Physical Activity Practice were used to assess physical activity level and barriers, respectively. No statistically significant difference was observed on the prevalence of physical inactivity in either gender or age groups. Regarding barriers, the proportion of 9 out of 22 barriers was statistically significant between men and women. Self-reported physical inactivity/activity in older Brazilian adults continues to be a concern. Uncommonly, older males reported a higher prevalence of physical inactivity compared to their counterparts. Additionally, physical inactivity prevalence continued to increase with the aging process. Yet, personal barriers such as lack of time and poor health were strongly associated with physical inactivity. The results of this study may help health professionals and public policy makers to better address the issues related to a healthy lifestyle among older adults and promote physical activity among Brazilian older adults and in other countries with similar characteristics.

## 1. Introduction

The benefits of physical activity have been widely published in the literature. The many advantages of physical activity include promoting health and well-being, delaying or preventing the onset of chronic disease and disability, and reducing mortality [1, 2]. Despite the well-known benefits of physical activity and the public health efforts to promote physical activity, physical inactivity remains a concern throughout the world [3, 4]. Compared to younger age groups, older adults present the highest prevalence of physical inactivity [5, 6]. For instance, Florindo and colleagues [6], examining the physical inactivity epidemiology in a Brazilian capital, observed that older adults present the highest prevalence of insufficient physical activity compared to their younger counterparts [6]. Similar findings can be observed in several other studies [5, 7–9].

 Several factors have been associated with decreased physical activity levels during the lifespan. Chronic diseases and other conditions frequently related to the aging process and poor health have been commonly reported as barriers to physical activity. For instance, Dawson and colleagues [10, 11] observed that around 53% of the individuals in their sample reported at least one barrier to engaging in walking, with poor health being the most cited. Similar findings were observed by Rasinaho and colleagues [12]. 

Physical activity and barriers to participation are strongly influenced by socioeconomic, demographic, environmental factors and exercise mode. Therefore, it is important to understand such behavior in different settings and contexts. For instance, Benedetti and colleagues [13] investigated physical activity and health conditions in a sample of 1,062 older adults living in Florianopolis, Brazil, either engaged or not engaged in social welfare groups. The authors observed no difference in the prevalence of physical inactivity (nearly 40%) for both groups. In addition, 74% of the participants reported illness; however the presence of illness was not associated with physical activity level (active and insufficient active). Siqueira and colleagues [14] explored physical activity level in over 4,000 older adults living in areas attended by primary healthcare units in the south and northeast of Brazil. The authors observed that 58% of their sample was considered physically inactive according to international recommendations (<150 minutes/week). Although using a large sample size and the same instrument and procedures to assess physical activity (IPAQ) the previous mentioned studies did not seek to include an instrument aiming to explore in depth possible barriers to physical activity. Reichert and colleagues [15] investigated barriers regarding adults in general (>18 years old); however the authors investigated only barriers related to leisure-time physical activity. Regarding older adults, it is known that all physical activity domains are important in order to achieve the current recommendation of 150 minutes/week [16]. In addition, most studies tend to collapse older individuals into only one category (e.g., 70 years and over or 65 years and over), which limits the findings about possible age differences. The purpose of this study was to examine the prevalence of physical inactivity and perceived barriers to physical activity in a community dwelling of older adults living in Brazil. Additionally, this study focused on different age groups and on gender in an attempt to determine whether or not age and gender modulate these variables. Physical activity behavior is a dynamic process influenced by many factors; therefore, the present study will be grounded in the social ecological theory of health behavior [17]. This theory suggests that individual behavior occurs in the interaction between the individuals and the environment and community in which they live [18]. 

Studies trying to identify factors associated with physical inactivity in different settings are important in order to address the wide array of differences between countries, environment, and individuals and for the development of strategies and interventions tailored specifically for each population. It is particularly important regarding the older adult population, which presents the highest prevalence of physical inactivity. Factors associated with physical activity barriers have received large attention from investigators in developed countries; however, Brazil is an emerging nation, where large transformations in the economy and health field have been seen in the last few years. The main perceived barriers for physical activity remain unclear in a developing country such as Brazil, especially regarding older adults. Studies, to date, that addressed this issued have used small samples, which limit the generalization of the findings. Therefore, the present study adds knowledge to current literature by investigating physical activity as a whole (occupation, transportation, housework, and leisure time) and related barriers in a representative sample of Brazilian older adults—using a broad instrument with 22 barrier-options and by attempting to analyze the participants in different older age groups.

## 2. Methods

A cross-sectional, population-based study was conducted in Rio Claro, São Paulo, Brazil, a midsized city (190,000 inhabitants) in central-southern Brazil. The physical activity level of the participants was measured using the long version of the International Physical Activity Questionnaire (IPAQ) [19], which is also validated for Brazilian older adults [20], and barriers to the practice of physical activity were assessed by means of a specially designed questionnaire [21]. Studies in the literature have recommended the use of accelerometers when measuring physical activity level, once it is a more direct measure, and can provide more reliable information. However, the use of accelerometers may not be afforded in all studies especially large-scale studies. Therefore, in order to accomplish our aim we conducted our investigation using the well-known IPAQ questionnaire, although aware of its limitations. The São Paulo State University Ethical Committee approved the research protocol, and informed consent was obtained from each subject before data collection.

## 3. Participants

A stratified random sampling procedure was used to select a representative sample of adults living in Rio Claro. From a total number of geographic census tracts (*n* = 200), half were randomly selected for inclusion in the study, and eight households within each tract were randomly chosen to be interviewed. This yielded a total of 800 households. All residents over 20 years of age in each home in the selected tracts, and who were able to walk independently, were eligible for inclusion in the study. This procedure resulted in a sample of 1,572 individuals who were interviewed. However, for the purposes of the present study, only the data related to older adults 60 years and over (*n* = 359) were analyzed. 

## 4. Measures

Physical activity level was assessed using the IPAQ long form, version 8. The IPAQ questionnaire estimates physical activity levels in four domains (occupation, transportation, housework, and leisure time). A total physical activity score was calculated following procedures reported by Hallal and colleagues [22] and used in several other different studies [6, 15, 22]. The score was calculated using the sum of the number of minutes of total moderate activity (including walking), plus two times the number of minutes of vigorous activity. The IPAQ defines moderate activity as an activity, performed for at least 10 minutes, that produces an increase in respiration and heart rate and causes sweating. Vigorous activities are defined as those activities that, performed for at least 10 minutes, produce even greater increases in breathing, heart rate, and sweating. For the purpose of this study, we defined insufficient physical activity in accordance with the Department of Health and Human Services of the United States Guidelines [16]. Individuals who reported less than 150 minutes per week of combined moderate and vigorous physical activity were considered to be insufficiently active. Similarly, people who reported at least 150 minutes per week of combined moderate and vigorous physical activity were considered to be active.

Barriers to physical activity engagement were assessed using the Questionnaire of Barriers to Physical Activity Practice (QBPAP) [21], which was developed based on barriers reported in scientific literature [23, 24]. This questionnaire was developed by Hirayama [21] to attend the purpose of his thesis. The first version of the QBPAP questionnaire was developed using the Likert scale and it was composed of 22 factors/barriers. According to the author, the Likert scale, was very difficult to understand by older adults, especially by clinical patients. To attend the purpose of the present study (epidemiologic approach) the QBPAP was modified in a list of 22 dichotomous (yes/no) factors and was previously tested in a pilot study. The pilot study consisted in the application of the QBPAP questionnaire in a sample of older adults living in a census tract sector not selected to be included in the study. After the pilot study, a few questions (barriers) were readjusted in order to provide better clarification. Following data collection, the internal consistency of the QBPAP questionnaire was tested by means of Cronbach's Alpha Coefficient, which returns 0.61 of standardized values, which means that the questionnaire met the conditions to be used for the proposed objective. 

## 5. Data Collection

Questionnaires were administered face to face by trained interviewers who had at least a high school education. The interviewers received 40 hours of training to correctly administer and code the questionnaires and were not aware of the aims of the study. The training sections were led by the investigators and used a manual developed specifically for this purpose, which included interview techniques with dramatizations about how to appropriately administer the questionnaire. The interviewers also were instructed with regard to terminology and how to interpret terms such as moderate and vigorous physical activity and were shown examples of common activities. Following the training procedures, a total of 17 interviewers were selected for data collection. 

Fieldwork supervisors (research staff) were responsible for checking the questionnaires completed by the interviewers. Fieldwork supervisors checked 40% of the questionnaires that each interviewer completed in order to ensure the reliability and validity of the data. They checked 30% by telephone and the remaining 10% by returning to the participant's home less than a week after the initial interview. The supervisors checked the questionnaires using a miniquestionnaire that was specifically developed for this purpose with key questions that were compared to the original questionnaires. When inconsistencies occurred, the interview was repeated by a different interviewer. This process identified inconsistencies in about 3% of the interviews, which were replicated.

After data collection the physical activity data was controlled for possible outliers. Such control was done by means of total day time (24 hours). According to the physical activity data provided, individuals that did not present at least 8 hours of sleep were excluded from the analysis with the assumption that they may overestimate their physical activity level. This procedure resulted in zero subjects excluded from the sample. 

## 6. Statistical Analysis

Statistical descriptive analyses (mean, standard deviation, and percentage) were performed. Differences between groups in the categorical variables were calculated using the chi-square for testing differences in proportions, *u* Mann Whitney, and Kruskal Wallis tests, at *P* < 0.05. Logistic regression was performed to observe a possible existence of association between physical inactivity and reported barriers adopting a 95% confidence interval.

## 7. Results

The final sample was comprised of 359 individuals, aged 60 years and over (females: *n* = 225, 62.7%; males: *n* = 134), with women constituting the majority even when the participants were distributed in age groups (Table 1). For the entire group of participants, the average body mass index was 26.2 kg/m^2^, which resulted in a classification as overweight. Around 65% reported good health; 58% were married, and 71% had completed high school.

**Figure 1:**
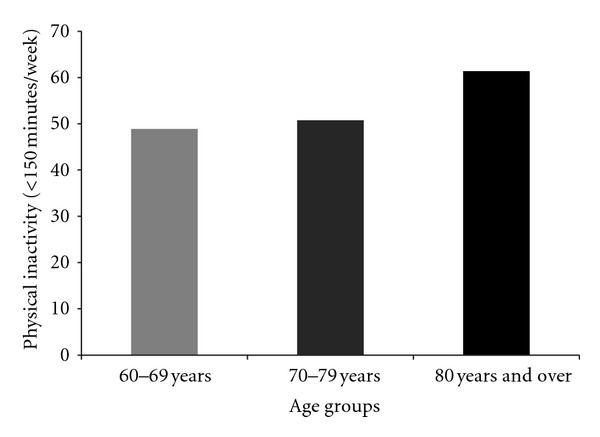
Prevalence of physical inactivity in Brazilian older adults according to age group.

In general 50.7% of the older adults interviewed did not meet the physical activity guidelines of 150 minutes per week of moderate physical activity and were considered insufficiently active. Examined by gender, men reported higher levels of physical inactivity (60.74%) compared to women (45.53%); however, such differences were not statistically significant (*χ* ^2^ = 1.867; df = 1; *P* = 0.17). Physical activity data were also compared between age groups. The oldest group (80 years and over) reported the highest prevalence of physical inactivity (61.36%) compared to their younger counterparts; however, the prevalence observed in the oldest age group was not statistically significant compared to others (*χ* ^2^ = 1.540; df = 2; *P* = 0.46). Details are presented in Figure 1.

Regarding barriers to physical activity, 100% of the participants, regardless of age group and gender, reported at least one barrier. The most frequently reported barriers for men were “Feel already active enough” and “need to relax during free time.” For women, “fear of falling and being hurt” and also “need to relax during free time” were among the most reported. The proportion of perception of barriers was significantly different (*P* < 0.05) between the men and women for nine out of 22 barriers (Table 2).

The mean number of barriers reported was significantly (*P* < 0.05) higher for women compared to men in the 60–69 year age groups and marginally significant in the 70–79 and 80 years and over groups (*P* = 0.08). Detailed information regarding the mean number of barriers is depicted in Table 3.

The perceptions of barriers were quite similar among older males regardless of age group. Notably, 91% of the oldest women group reported barriers associated with a lack of environmental safety. 

Although 22 barriers were investigated in the present sample, only poor health and fear of injury were positively associated with physical inactivity, whilst being active enough was negatively associated with the outcome. The associations remain after adequate adjustments.  Table 4 displays the associations observed.

## 8. Discussion

This study sought to investigate physical inactivity rates and related barriers in older adults living in the city of Rio Claro, São Paulo, Brazil. To our knowledge the study is the first in Brazil using a representative sample of older adults addressing barriers and physical activity. Previous investigations in Brazil using a representative sample of older adults did not include an instrument to assess barriers in depth [13, 14], and studies using adults in general include older adults just as a subset in their analysis [15].

The present findings revealed that older adults living in Rio Claro, Brazil, reported high levels of physical inactivity (50.7%). Additionally, increased physical inactivity levels tended to increase through age groups (please report to Figure 1). Regarding gender, men in our study reported higher levels of physical inactivity compared to their counterparts; however differences were not statistically significant. These findings are in line with the literature [3, 22, 25, 26]. For example, Knuth and colleagues [25] investigated changes in physical activity over a five-year period in adults using the short version of the IPAQ questionnaire. In general, the authors observed an increase in physical inactivity among the participants (41 to 52%). Regarding older adults, Knuth and colleagues [25] observed higher prevalence of physical inactivity compared to our study. Despite differences in prevalence, both studies observed that physical inactivity increases as a function of the aging process. Regarding gender differences in physical activity level, Hallal and colleagues [22] verified that women in their study presented lower levels of physical inactivity compared to men. Similar findings were also observed in our study. According to Hallal and colleagues [22] household-related physical activity is an important part of the total amount of physical activity for women. In the present study only 18% of the women reported zero minutes per week in household-related physical activity compared to 42% of men. Such common patterns in Latin America regarding household tasks appear to play an important role in the total physical activity for women. 

Regarding physical activity barriers, 100% of the assessed sample reported at least one barrier to engaging in physical activity. This finding differs somewhat from those reported by Reichert and colleagues [15] and Dawson and colleagues [10, 11]. Of the mentioned studies, 85% and 53% of the samples, respectively, reported at least one barrier. Possibly, methodological differences in both studies could explain, at least in part, the different findings. Reichert and colleagues [15], for example, assessed individuals 20 years of age and older; therefore, the younger population might be counted to reduce the prevalence of barriers observed. Younger populations appear to have different perceptions of health compared to older adults [27] and generally have wider social networks as well. The current social emphasis on the physical esthetic helps motivate young people to be engaged in physical activities. A lower prevalence of barriers in the Dawson et al. study [10, 11] may be explained by the fact that the authors focus only on barriers related to walking. However, the present study assessed barriers that could be related to any kind of physical activity. 

Differently, Stathi and colleagues [28] performing a mixed method to assess physical activity and barriers in older adults (70 years and over) observed functional limitations, lack of intrinsic motivation, and not having an activity companion as the highest impact on barriers in their study. Albeit intrinsic motivation was not included in our questionnaire, our sample revealed high prevalence in the barriers “need to relax,” “too lazy,” and “lack of energy.” These barriers are strongly related to lack of motivation [29, 30]. In addition, the barrier need to relax during the free time, reveals that older Brazilian adults may think physical activity in some sort of “old fashion” types of exercises when severe intensity would be required. Nowadays, exercise may also be performed as a way of relaxation. It could be the cause of lack of health literacy or even the fact that as older adults they have done a lot of physical activity throughout their lives and they believe no more is needed, as most of them reported to be already physically active enough. Regarding functional limitation, it was included in our study as “disease or disability,” which was reported by 50% of men and 69% of women in the oldest age group. Interestingly, not having an activity companion, described in our study as “lack of company,” appears as an important barrier to the youngest old group (60–69 years old). The prevalence of the barrier, “fear of injury,” seems noteworthy. Despite the strong association observed in the present study, 91% of the women in the 80-year-old group reported it as a barrier. Such prevalence suggests either a self-perception of frailty or a reduced self-efficacy in this age group. The barrier “active enough” appears to affect approximately 53% of men and women (60 to 79 years old), however, not the oldest group. In fact, this barrier revealed to be negatively associated with physical inactivity. It may suggest a false positive perception of being active for health benefits. According to Costello and colleagues [31], people who are physically inactive perceived themselves to be physically active, mostly due to their perception of physical activity being grounded in a social context, which may not represent a real physical activity behavior that benefits health. That is a concern, because compelling evidence demonstrated that health benefits from physical activity are achieved if the individual performs at least 150 minutes per week of moderate physical activity [32]. If physically inactive persons' perception of physical activity is grounded in social context, public health makers should attempt for this aspect to develop public policies, strategies, and interventions, and health professionals need to be aware of the role that social context background has in older adults in order to recommend physical activity.

In general, there are many similarities in the barriers, both for men and women, regardless of age groups. Barriers like “need to relax” and “lack of energy,” which are strongly related to lack of motivation, were common in our sample. Additionally, these barriers are comparable to barriers related to health status, such as “poor health” and “have a disease or disability.” These findings suggest that public health strategies and interventions should address programs to improve self-efficacy and programs to empower older adults to self-manage their disease and disabilities, which can highly contribute to frailty. These findings also can support health professional's use of physical activity as part of the treatment plan. 

The high prevalence of physical inactivity observed in the present study is a public health concern due to the well-known negative effects of physical inactivity. Despite the negative effects on physical and mental health, and consecutively quality of life, physical inactivity is an economic burden to the government [33, 34]. For countries in which health services are supported by the government, for example, Brazil, Canada, and France, it is extremely important. Billions of dollars can be saved per year if the population increased their level of physical activity. Moreover, compelling evidence in the literature clearly states the benefits of physical activity for older adults [1]. 

To our knowledge, the present study is the first population-based investigation of older adults, and therefore, producing more robust evidence regarding physical activity and barriers of this population in Brazil. These findings have enabled us to develop a better insight of older adult's physical inactivity and a better understanding of the obstacles that prevent older adults from engaging in physical activity on a regular basis in an emerging country. The incidence of diseases in older adults can be worsened by physical inactivity, and overall health may be improved by regular participation in physical activity. Taken together, the findings of the present and similar studies can be a support for the development of public health policies and strategies, specifically tailored for older adults. In addition, these findings can support health professionals, such as physicians, if physical activity is addressed during medical consultation. 

Rio Claro presents, relatively, one of the largest older adult populations in Brazil, being 13.5% aged 60 years and over [35]. The findings of the study, combined with the population characteristics, provide an opportunity for public health interventions in Rio Claro. The generalization of these findings may be questionable, because Rio Claro presents some characteristics that most other Brazilian cities do not have (e.g., inner midsized city 112 miles from the capital São Paulo; one campus of a top Brazilian University; Rio Claro was the first city to elaborate a specific elderly county law, etc.). Another important aspect of the present findings, in terms of public health, lies in the lack of national data on this topic. Brazil has one national survey dedicated to monitoring health behaviors (physical activity, alcohol consumption, food intake, prevalence of obesity, type 2 diabetes, etc.), named “Vigilância de Fatores de Risco e Proteção para Doenças Crônicas por Inquérito Telefônico.” The first publication of the survey occurred in 2002 and has been published every two years since then. Although important, the survey does not provide any information about possible barriers that could play a role in physical inactivity. Additionally, the survey takes place only in the capitals, which presents an entirely different dynamic compared to inner cities, especially regarding behaviors.

Albeit important, our findings should be interpreted with caution due to study limitations. First, we use a cross-sectional design, and it does not provide information on causal effect. Additionally, we divided our sample by age group, and the oldest-old group (80 years and over), especially for men, yielded only 12 individuals, which could bias the results. In addition, we assessed physical activity level using a self-reported physical activity questionnaire. Although the IPAQ questionnaire is a valid instrument and used worldwide, some studies have reported concerns about its accuracy in estimating physical activity level [36, 37], due to problems related to overestimation of the results. Therefore, although the prevalence of physical inactivity observed in our study is in consonance with the literature, it is important to highlight that our sample was found to be quite active (generally nearly 50% was considered active for health benefits; physical activity ≥ 150 minutes/week), which probably reflects the difficulties associated with the use of IPAQ questionnaire to accurately assess physical activity levels. Thus, such information supports the idea that although the IPAQ is validated for different countries, some sort of cultural validation is still needed to improve the sensibility of such an instrument to measure physical activity among populations. Furthermore, although we controlled the IPAQ data for outliers using a daytime cut-off, as aforementioned, we did not verify our data based on more accurate statistical procedures (Skews and kurtosis), so possible outliers could exist in our sample. Future studies should attempt, whenever possible, for the use of more direct measures, such as accelerometry. Despite the criticism related to the use of the IPAQ questionnaire, useful information can be drawn from the data provided. It is important to mention that this study is part of a larger one that was conducted in the city of Rio Claro, São Paulo, Brazil, where we aim to investigate the prevalence of physical inactivity and associated factors in adults over the age of 18 years. Therefore, as the first large study that took place in the city, this data represents the first “snapshot” of the physical inactivity prevalence of the adult population. In such case the use of self-reported questionnaire is understandable, as in the cases of monitoring, such as in national surveillances. However, when using self-reported information related to physical activity, by means of IPAQ, or other ones, it should be advised to include other studies results and also with different health information before proceeding with interventions and strategies to promote tailored physical activity. Second, although we used a large barrier questionnaire, it certainly does not cover all possible barriers that may exist, especially regarding environmental barriers. The present study included only three possibilities. It is possible that participants have other barriers that were not addressed. Thereby, studies using a mixed method design (qualitative and quantitative), in different settings, are important throughout in order to monitor both physical activity level and perceived barriers to physical activity for older adults in order to provide strategies and interventions if needed. 

In short, a high prevalence of physical inactivity was observed in older Brazilian adults living in a midsized city. It was observed that men reported higher levels of physical inactivity compared to women. Additionally, physical inactivity keeps increasing as adults age after 60. Consistent with these findings, high prevalence of barriers was reported by the participants, with those participants 80 years and over reporting significantly greater numbers of barriers than younger age groups. Despite the lower physical inactivity level compared to men, women reported significantly higher numbers of barriers compared to their counterparts. This suggests that, although perceiving greater number of barriers compared to men, women are more likely to overcome these barriers and be active, or the barriers reported by women in the present study are more temporal. Regarding physical activity barriers, “poor health,” “have a disease and disability,” low self-efficacy, diagnosed by “fear of injury” and barriers related to lack of motivation were mostly reported. Yet, despite the large number of barriers addressed, only poor health and fear of injury were positively associated with physical inactivity. 

These findings should be a concern to the government and public health policy makers, due to the negative effects of physical inactivity in physical and mental health and economic costs. Several studies have demonstrated a strong association between physical inactivity and chronic diseases, such as type two diabetes, hypertension, and cognitive decline. Therefore, the results of this study may help public policy makers to better address the issues of active lifestyle among the older population and promote physical activity.

## Figures and Tables

**Table 1:** Number of individuals, mean age, and standard deviation of older men and women, in different age groups, Rio Claro, São Paulo, Brazil.

Age groups (years)	Men		Women	
	*N*	Mean ± SD (years)	*N*	Mean ± SD (years)
60–69	69	64 ± 3.07	108	64 ± 2.64
70–79	53	74 ± 2.81	85	74 ± 2.97
≥80	12	83 ± 3.29	32	84 ± 4.51

**Table 2:** Barriers to being engaged in physical activity as reported by older Brazilians, according to gender, Rio Claro, São Paulo, Brazil.

Barriers	Men (*n* = 134) *n* (%)	Women (*n* = 225) *n* (%)	*χ* ^2^	*P*
Lack of time	37 (27.8)	81 (36.2)	2.624	0.10
Feel active enough	80 (59.7)	107 (48.2)	4.434	0.03
Lack of company	38 (28.4)	92 (40.9)	5.709	0.02
Lack of money	53 (39.6)	93 (41.7)	0.160	0.68
Too old	30 (22.4)	59 (26.2)	0.662	0.41
Have a disease	30 (22.6)	86 (38.2)	9.366	0.02
Poor health	21 (15.7)	52 (23.1)	2.869	0.09
Too shy	26 (19.4)	76 (33.9)	8.683	0.03
Bad experience	11 (8.2)	26 (11.6)	1.045	0.30
Lack of nearby facilities	56 (41.8)	91 (41.0)	1.139	0.56
Need to relax	75 (56.4)	129 (58.1)	0.100	0.75
Lazy	56 (41.8)	99 (44.0)	0.167	0.68
Fear of injury	34 (25.6)	124 (55.1)	29.598	0.01
Dislike exercise	43 (32.1)	69 (30.9)	0.051	0.82
Lack of appropriate clothes	27 (20.6)	36 (16.1)	1.126	0.30
Give up soon	37 (28.0)	72 (32.1)	1.298	0.52
Feel too fat/thin	21 (15.7)	52 (23.3)	3.009	0.01
Lack of energy	34 (25.6)	94 (41.8)	9.566	0.02
Do not believe in physical activity	1 (0.7)	6 (2.7)	1.646	0.20
Lack of security	33 (24.6)	79 (35.3)	4.416	0.04
Bad weather	42 (32.1)	99 (44.8)	5.556	0.02
Urinary incontinence	6 (4.5)	21 (9.5)	2.936	0.08

**Table 3:** Number of barriers (means) reported by older adults, by gender, and by age groups, Rio Claro, São Paulo, Brazil.

Age group (years)	Men	Women	*P* *U*MW
60–69	5.3^a^	6.5^d^	0.01
70–79	6.8^b^	7.8^e^	0.08
80-and-over	6.8^c^	9.3^f^	0.05

*U*MW*: U* Mann-Whitney test. Kruskal Wallis test: (a) significantly different from (b) (*P* < 0.02); (d) significantly different from (f) (*P* < 0.01) and (e) (*P* < 0.04); (e) significantly different from (f) (*P* < 0.015).

**Table 4:** Multiple logistic regression. Barriers to physical activity and insufficient physical activity.

Barriers	Crude analysis OR (IC 95%)	*P*	Adjusted analysis* OR (IC 95%)	*P*
Lack of time	0.78 (0.637–0.966)	0.022	0.81 (0.649–1.012)	0.063
Feel enough active**	0.74 (0.604–0.910)	0.004	0.75 (0.601–0.944)	0.014
Poor health	1.98 (1.351–2.909)	0.001	1.69 (1.067–2.702)	0.026
Fear of injury	1.49 (1.154–1.936)	0.002	1.37 (1.020–1.852)	0.037

*Adjusted for socioeconomic variables and all other barriers.

**Protection factor for physical inactivity.

## References

[1] Chodzko-Zajko WJ, Proctor DN, Fiatarone Singh MA (2009). Exercise and physical activity for older adults. *Medicine and Science in Sports and Exercise*.

[2] Hubert HB, Bloch DA, Oehlert JW, Fries JF (2002). Lifestyle habits and compression of morbidity. *Journals of Gerontology A*.

[3] Dumith SC, Hallal PC, Reis RS, Kohl HW (2011). Worldwide prevalence of physical inactivity and its association with human development index in 76 countries. *Preventive Medicine*.

[4] Guthold R, Ono T, Strong KL, Chatterji S, Morabia A (2008). Worldwide variability in physical inactivity. A 51-country survey. *American Journal of Preventive Medicine*.

[5] Zhao G, Ford ES, Li C, Balluz LS (2011). Physical activity in U.S. Older adults with diabetes mellitus: prevalence and correlates of meeting physical activity recommendations. *Journal of the American Geriatrics Society*.

[6] Florindo AA, Guimarães VV, Cesar CLG, De Azevedo Barros MB, Alves MCGP, Goldbaum M (2009). Epidemiology of leisure, transportation, occupational, and household physical activity: prevalence and associated factors. *Journal of Physical Activity and Health*.

[7] Shibata A, Oka K, Harada K, Nakamura Y, Muraoka I (2009). Psychological, social, and environmental factors to meeting physical activity recommendations among Japanese adults. *International Journal of Behavioral Nutrition and Physical Activity*.

[8] Marshall SJ, Jones DA, Ainsworth BE, Reis JP, Levy SS, Macera CA (2007). Race/ethnicity, social class, and leisure-time physical inactivity. *Medicine and Science in Sports and Exercise*.

[9] Sims J, Hill K, Davidson S, Gunn J, Huang N (2007). A snapshot of the prevalence of physical activity amongst older, community dwelling people in Victoria, Australia: patterns across the “young-old” and ‘fold-old’. *BMC Geriatrics*.

[10] Dawson J, Hillsdon M, Boller I, Foster C (2007). Perceived barriers to walking in the neighbourhood environment and change in physical activity levels over 12 months. *British Journal of Sports Medicine*.

[11] Dawson J, Hillsdon M, Boller I, Foster C (2007). Perceived barriers to walking in the neighborhood environment: a survey of middle-aged and older adults. *Journal of Aging and Physical Activity*.

[12] Rasinaho M, Hirvensalo M, Leinonen R, Lintunen T, Rantanen T (2007). Motives for and barriers to physical activity among older adults with mobility limitations. *Journal of Aging and Physical Activity*.

[13] Benedetti TRB, d'Orsi E, Schwingel A, Chodzko-Zajko WJ (2012). Convivência groups: building active and healthy communities of older adults in Brazil. *Journal of Aging Research*.

[14] Siqueira FV, Facchini LA, Piccini RX (2008). Physical activity in young adults and the elderly in areas covered by primary health care units in municipalities in the South and Northeast of Brazil. *Cadernos de Saude Publica*.

[15] Reichert FF, Barros AJD, Domingues MR, Hallal PC (2007). The role of perceived personal barriers to engagement in leisure-time physical activity. *American Journal of Public Health*.

[17] Stokols D, Allen J, Bellingham RL (1996). The social ecology of health promotion: implications for research and practice. *American Journal of Health Promotion*.

[18] Sallis JF, Cervero RB, Ascher W, Henderson KA, Kraft MK, Kerr J (2006). An ecological approach to creating active living communities. *Annual Review of Public Health*.

[19] Craig CL, Marshall AL, Sjöström M (2003). International physical activity questionnaire: 12-Country reliability and validity. *Medicine and Science in Sports and Exercise*.

[20] Benedetti TRB, Antunes PDC, Rodriguez-Añez CR, Mazo GZ, Petroski EL (2007). Reproducibility and validity of the International Physical Activity Questionnaire (IPAQ) in elderly men. *Revista Brasileira de Medicina do Esporte*.

[21] Hirayama MS Physical Activity and Parkinson disease: behavior change, self-efficacy and perceived barriers.

[22] Hallal PC, Victora CG, Wells JCK, Lima RC (2003). Physical inactivity: prevalence and associated variables in Brazilian adults. *Medicine and Science in Sports and Exercise*.

[23] Booth ML, Bauman A, Owen N, Gore CJ (1997). Physical activity preferences, preferred sources of assistance, and perceived barriers to increased activity among physically inactive Australians. *Preventive Medicine*.

[24] Brown WJ, Miller YD (2001). Too wet to exercise? Leaking urine as a barrier to physical activity in women. *Journal of Science and Medicine in Sport*.

[25] Knuth AG, Bacchieri G, Victora CG, Hallal PC (2010). Changes in physical activity among Brazilian adults over a 5-year period. *Journal of Epidemiology and Community Health*.

[26] Shibata A, Oka K, Nakamura Y, Muraoka I (2009). Prevalence and demographic correlates of meeting the physical activity recommendation among Japanese adults. *Journal of Physical Activity and Health*.

[27] Idler EL, Benyamini Y (1997). Self-rated health and mortality: a review of twenty-seven community studies. *Journal of Health and Social Behavior*.

[28] Stathi A, Gilbert H, Fox KR, Coulson J, Davis M, Thompson JL (2012). Determinants of neighborhood activity of adults aged 70 and over: a mixed methods study. *Journal of Aging and Physical Activity*.

[29] Bowles HR, Morrow JR, Leonard BL, Hawkins M, Couzelis PM (2002). The association between physical activity behavior and commonly reported barriers in a worksite population. *Research Quarterly for Exercise and Sport*.

[30] Trost SG, Owen N, Bauman AE, Sallis JF, Brown W (2002). Correlates of adults' participation in physical activity: review and update. *Medicine and Science in Sports and Exercise*.

[31] Costello E, Kafchinski M, Vrazel J, Sullivan P (2011). Motivators, barriers, and beliefs regarding physical activity in an older adult population. *Journal of Geriatrics and Physical Therapy*.

[32] Hallal PC, Matsudo SM, Matsudo VK, Araújo TL, Andrade DR, Bertoldi AD (2005). Physical activity in adults from two Brazilian areas: similarities and differences. *Cadernos de Saude Publica*.

[33] Anderson LH, Martinson BC, Crain AL (2005). Health care charges associated with physical inactivity, overweight, and obesity. *Preventing Chronic Disease*.

[34] Pate RR, Pratt M, Blair SN (1995). Physical activity and public health: a recommendation from the Centers for Disease Control and Prevention and the American College of Sports Medicine. *Journal of the American Medical Association*.

[35] http://www.ibge.gov.br/cidadesat/topwindow.htm.

[36] Hallal PC, Gomez LF, Parra DC (2010). Lessons learned after 10 years of IPAQ use in Brazil and Colombia. *Journal of Physical Activity & Health*.

[37] Rzewnicki R, Vanden Auweele Y, De Bourdeaudhuij I (2003). Addressing overreporting on the International Physical Activity Questionnaire (IPAQ) telephone survey with a population sample. *Public Health Nutrition*.

